# Transcriptional analysis of peripheral memory T cells reveals Parkinson’s disease-specific gene signatures

**DOI:** 10.1038/s41531-022-00282-2

**Published:** 2022-03-21

**Authors:** Rekha Dhanwani, João Rodrigues Lima-Junior, Ashu Sethi, John Pham, Gregory Williams, April Frazier, Yaqian Xu, Amy W. Amara, David G. Standaert, Jennifer G. Goldman, Irene Litvan, Roy N. Alcalay, Bjoern Peters, David Sulzer, Cecilia S. Lindestam Arlehamn, Alessandro Sette

**Affiliations:** 1 Division of Vaccine Discovery, La Jolla Institute for Immunology, La Jolla, CA 92037 USA; 2 Aligning Science Across Parkinson’s (ASAP) Collaborative Research Network, Chevy Chase, MD USA; 3 Department of Neurology, Columbia University, Division of Molecular Therapeutics, New York State Psychiatric Institute, New York, NY 10032 USA; 4 Department of Neurology, University of Alabama at Birmingham, Birmingham, AL 35233 USA; 5 Shirley Ryan AbilityLab, Northwestern University Feinberg School of Medicine, Chicago, IL 60611 USA; 6 Department of Neuroscience, University of California San Diego, La Jolla, CA 92093 USA; 7 Department of Neurology, Columbia University Irving Medical Center, New York, NY 10032 USA; 8 Department of Medicine, University of California San Diego, La Jolla, CA 92093 USA; 9 Departments of Psychiatry and Pharmacology, Columbia University, New York State Psychiatric Institute, New York, NY 10032 USA

**Keywords:** Neuroimmunology, Neuroimmunology

## Abstract

Parkinson’s disease (PD) is a multi-stage neurodegenerative disorder with largely unknown etiology. Recent findings have identified PD-associated autoimmune features including roles for T cells. To further characterize the role of T cells in PD, we performed RNA sequencing on PBMC and peripheral CD4 and CD8 memory T cell subsets derived from PD patients and age-matched healthy controls. When the groups were stratified by their T cell responsiveness to alpha-synuclein (α-syn) as a proxy for an ongoing inflammatory autoimmune response, the study revealed a broad differential gene expression profile in memory T cell subsets and a specific PD associated gene signature. We identified significant enrichment of transcriptomic signatures previously associated with PD, including for oxidative stress, phosphorylation, autophagy of mitochondria, cholesterol metabolism and inflammation, and the chemokine signaling proteins CX3CR1, CCR5, and CCR1. In addition, we identified genes in these peripheral cells that have previously been shown to be involved in PD pathogenesis and expressed in neurons, such as LRRK2, LAMP3, and aquaporin. Together, these findings suggest that features of circulating T cells with α-syn-specific responses in PD patients provide insights into the interactive processes that occur during PD pathogenesis and suggest potential intervention targets.

## Introduction

Parkinson’s disease (PD) is a progressive neurodegenerative disorder characterized by two hallmarks: (i) loss of dopaminergic neurons in the substantia nigra (SN) of the brain responsible for the motor features^1^ and (ii) excess accumulation of aggregated α-synuclein (α-syn) protein^2^. This loss of dopaminergic neurons in the SN is believed to be the reason for the parkinsonian motor signs (increased rigidity, slowness, rest tremor, and at later stages postural instability) observed in PD^3^. There are approximately 1 million people in North America affected by this debilitating disease^4^. The diagnosis and management of PD are challenging as the disease is constrained by limited treatment options, which are mainly focused on improving postural instability and non-motor (constipation, mood, sleep, and cognition) symptoms. Considering the increasing prevalence and overall societal impact of PD, it is imperative to explore the underlying mechanisms that play a role in the progression of this heterogeneous and complex disease and ultimately to develop targeted symptomatic and disease-modifying interventions.

Several lines of evidence highlight an association of PD with inflammation. In 1988, a landmark postmortem study by McGeer and colleagues reported activated microglia in SN of PD subjects^5^. Since then, several reports and studies have indicated an association between an enhanced inflammatory response and PD^6^. More recent studies have revealed an autoimmune component in PD, which comprises recognition of several α-syn-derived T cell epitopes by CD4 T cells^7^, and demonstrate an increased α-syn-specific T cell reactivity in preclinical and early stages of the disease^8^.

These observations can be interpreted in the broader context of the current understanding of PD pathogenesis and progression. It is widely thought that clinically diagnosed motor PD and cognitive impairment is preceded by a long (often decades) prodromal phase, associated with symptoms ranging from alteration of the sense of smell, constipation, and sleep disorders that may precede the loss of SN dopaminergic neurons^9^. Indeed, in a single case study where T cell samples were available years prior to and after disease onset, α-syn-specific CD4 T cells were detected at higher levels before disease symptomatic onset^8^.

To define molecular alterations associated with PD, we compared the transcriptional profiles of peripheral T cells derived from individuals with diagnosed motor PD to those of age-matched healthy controls (HC). We hypothesized that PD patients exhibiting α-syn-specific T cell responses (PD Responders; PD_R) are associated with an inflammatory stage of the disease, while the non-responder category (PD_NR) is associated with a non-α-syn-specific and/or later stage when inflammatory features of the disease have subsided. We have identified differences in transcriptomic profiles associated with both CD4 and CD8 T cell subsets that are apparent if the PD subjects are classified based on T cell reactivity to α-syn (PD_R vs. PD_NR). The results indicate that genes differentially regulated in CD4 memory T cells were enriched in oxidative stress and autophagy functions, while those upregulated in CD8 memory T cells were enriched in inflammatory and chemotaxis-related gene functions.

## Results

### Classification of PD subjects based on α-syn specific T cell reactivity

In previous studies, we detected α-syn specific T cell responses in approximately 40–50% of PD subjects^7,8^. We further reported that α-syn specific T cell reactivity is specifically associated with preclinical and timepoints <10 years since diagnosis (prior to sample donation) following the onset of motor PD features^8^, while responses subsided in later stages of PD. Based on this finding, we hypothesized that PD subjects that demonstrate α-syn-specific T cell reactivity could be a “proxy” for individuals associated with an active inflammatory autoimmunity phenotype, and that analysis might reveal a transcriptional profile distinct from subjects without PD (HC) or PD subjects that do not exhibit α-syn T cell reactivity.

Accordingly, based on the magnitude of total response mounted against α-syn peptides, PD subjects were classified in two categories: responders (denoted as PD_R; > 250 SFC for the sum of IFNγ, IL-5, and IL-10) and non-responders (denoted as PD_NR; < 250 SFC). IFNγ, IL-5, and IL-10 were chosen as markers of T cell reactivity as they capture a broad immune response (i.e., Th1/Th2/Treg) and we have previously shown them to be detected at higher levels in PD^7,8^. We also included age-matched HC who were α-syn non-responders (HC_NR), to avoid the possibility that HC who exhibit α-syn-specific T cell reactivity may be in prodromal stages of PD. The classification criteria were based on our previously published studies^7,8^ where we determined α-syn-specific T cell reactivity for PD following in vitro restimulation assays and measured cytokine release by Fluorospot or ELISPOT assays.

**Fig. 1:**
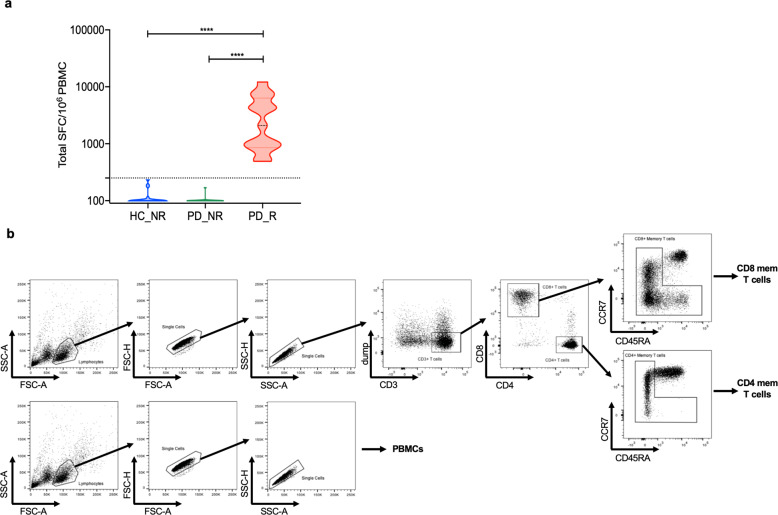
Classification of PD and age-matched HC based on the α-syn T cell response. **a** Violin plot shows the magnitude of T cell response (sum of IFN-γ, IL-5, and IL-10) in HC non-responders (HC_NR) (*n* = 20) PD responders (PD_R) (*n* = 15), and PD non-responders (PD_NR) (*n* = 21). The dotted line denotes the cut-off value of 250 SFC. Two-tailed Mann–Whitney, ****p < 0.0001. **b** The gating strategy was adopted to identify and sort PBMC, CD4, and CD8 memory T cells from PD and HC subjects.

To investigate differential gene expression signatures, we examined 34 PD subjects including PD_R (*n* = 14) and PD_NR (*n* = 20) (Fig. 1a). For control subjects, we selected 19 HC_NR subjects. We first analyzed the relative frequency of major PBMC subsets, i.e., monocytes, NK cells, B cells, T cells, and CD4 and CD8 memory T cells by flow cytometric analysis. The frequency of each PBMC subset was remarkably similar in all groups (Supplementary Fig. 1A) and there was no significant difference between CD4 and CD8 memory T cell subsets (Supplementary Fig. 1B, C).

### Transcriptional analysis of PBMC, CD4, and CD8 memory T cells in PD and age-matched HC

We then examined the hypothesis that the circulating peripheral lymphocytes reflect a general inflammatory state associated with PD < 10 years since diagnosis. We analyzed PBMC, CD4, and CD8 memory T cells from PD_R, PD_NR, and HC_NR subjects for specific transcriptomic signatures that might be associated with PD. The low frequency of α-syn-specific CD4 T cells detected in PBMCs in PD^7,8^ requires 2 weeks in vitro culture to produce sufficient cells for characterization. CD4 and CD8 memory T cell subsets were identified using CCR7 and CD45RA immunolabel and were sorted based on the gating strategy in Fig. 1b. Whole PBMC and sorted CD4 and CD8 memory T cell populations were sequenced with the Smart seq protocol^10^. To assess whether differences in gene expression could distinguish the groups, we applied principal component analysis (PCA). As expected, the global gene expression profile analyzed by PCA revealed three distinct clusters corresponding to the PBMC, memory CD4, and memory CD8 T cell subsets. However, the same analysis did not discriminate between the PBMC, CD4, or CD8 memory T cells from PD and HC subjects (Supplementary Fig. 2A).

We next performed differential gene expression analysis (DEseq) comparing PD vs. HC_NR to explore PD-specific gene expression signatures of PBMC, CD4, and CD8 memory T cells (see RNA-seq analysis methods for data availability). Only 26 genes were differentially expressed in PBMC between PD and HC_NR [fold change ≥1.5 (absolute log2 ≥ 0.58) and adjusted p-value <0.05]. Of the 26 genes, only 18 were protein-coding; 7 were upregulated and 11 downregulated. (Table 1). A total of 11 genes (1 upregulated and 10 downregulated; Table 1) and 9 genes (4 upregulated and 5 genes downregulated; Table 1) were differentially expressed protein-coding genes in CD4 and CD8 memory T cells, respectively. In conclusion, few genes were differentially expressed at the global level, and we did not identify any specific molecular pathway that was differentially regulated in PBMC, CD4, or CD8 memory T cells. Moreover, no overlap was observed between the few protein-coding genes that were differentially expressed in PD vs. HC_NR, in PBMC, CD4, or CD8 cell subsets (Supplementary Fig. 2B).

**Table 1:** Number of differentially expressed genes in different comparisons.

Condition^a^	Cell type	DE protein coding genes		
		Up	Down	Total
PD vs. HC_NR	PBMC	7	11	18
	CD4	1	10	11
	CD8	4	5	9
PD_R vs. PD_NR	PBMC	18	72	90
	CD4	168	136	304
	CD8	284	49	333
PD_R vs. HC_NR	PBMC	19	46	65
	CD4	81	91	172
	CD8	192	35	227

^a^*PD* Parkinson’s disease; *PD_R* PD responders to α-syn; *PD_NR* PD non-responders; *HC_NR* healthy control non-responders.

### Classification of PD subjects based on α-syn-specific T cell reactivity reveals specific gene signatures

**Fig. 2:**
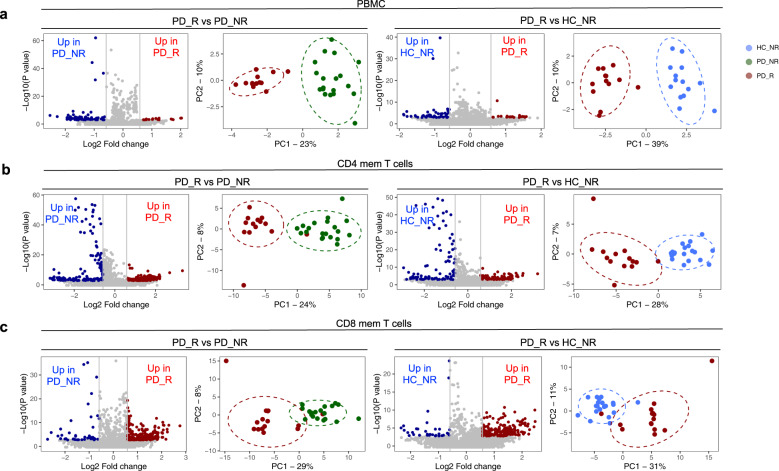
α-syn specific T cell reactivity is associated with a unique gene expression profile. Volcano plots show log_2_ fold change vs. −log_10_(*P* value) for the PD_R (*n* = 15) vs. PD_NR (*n* = 21) and PD_R vs. HC_NR (*n* = 20), respectively. The subset of genes with an absolute log2 fold change >1.5 and adjusted *p*-value less than 0.05 were considered significant and are indicated by dotted lines. Red dots of volcano plots indicate protein-coding genes upregulated in PD_R and blue dots indicate protein-coding genes downregulated in PD_NR or HC_NR. PCA plots show distinct clusters of PD_R, PD_NR and HC_NR (**a**) PBMC (**b**) CD4 memory T cells (**c**) CD8 memory T cells based on differentially expressed protein-coding genes.

Next, we compared the gene expression profiles of PD_R to HC_NR and to PD_NR subjects. We observed a large increase in the number of differentially expressed genes in comparisons of each cell type (PBMC, CD4, and CD8 memory T cells; Table 1). The total number of differentially expressed genes for PBMC between PD_R vs. PD_NR and PD_R vs. HC_NR was 90 and 65, respectively (Fig. 2a). Scrutiny of these genes did not reveal any functional enrichment for specific patterns or pathways (Supplementary Table 1).

In contrast, CD4 and CD8 memory T cells exhibited an intriguing gene signature with an approximately ~2.5–4-fold increase in the number of differentially expressed genes between the PD_R and PD_NR groups and between PD_R and HC_NR. PD_R to PD_NR comparison revealed 304 DE genes for CD4 (136 downregulated and 168 upregulated; Fig. 2b), and 333 DE genes for CD8 (49 downregulated and 284 upregulated, Fig. 2c, Table 1). Similarly, comparing PD_R to HC_NR, revealed 172 DE genes for CD4 (91 downregulated and 81 upregulated, Fig. 2b), and 227 DE genes for CD8 (35 downregulated and 192 upregulated; Fig. 2c and Table 1). As expected, based on the DE genes, the disease groups PD_R, HC_NR, and PD_NR formed distinct clusters (Fig. 2). There was a substantial overlap of DE genes between PD_R vs. PD_NR and PD_R vs. HC_NR within each cell type, but minimal to no overlap of DE genes across different cell types (Supplementary Table 1). We have also analyzed the differentially expressed genes between PD_NR vs. HC_NR in PBMC, CD4, and CD8 T cell populations. We found 31, 55, and 49 DE genes meeting our cutoff criteria respectively in those groups, which is a much smaller number of genes compared to the comparisons with PD-R. We did not further pursue these analyses, as our study was more focused on the gene signature associated with individuals who have ongoing autoimmunity toward a-synuclein compared to those who do not.

Interestingly, PRKN and LRRK2 genes that are well established to be related to familial PD^11–17^, were differentially expressed in CD4 and CD8 memory T cells with both genes downregulated in CD4 and upregulated in CD8 memory T cells in PD_R compared to PD_NR and HC_NR, respectively (PRKN is up in PD_R vs. PD_NR: LRRK2 is up in PD_R vs. HC_NR) indicating that the two cell types play distinct roles in PD-associated T cell autoimmunity. We also identified differentially expressed genes including TFEB and UBAP1L that have been implicated in autophagy and ubiquitination^18^ in CD4 memory T cells. Notably, TFEB has been proposed to be a therapeutic target in PD^19–21^. Other PD related genes that we found to be differentially expressed between PD_R vs. PD/HC_NR included SEPT5 (down compared to CD4 PD_NR and HC_NR), GFRA2 (up compared to CD8 PD_NR and HC_NR), MAOA (down compared to CD8 HC_NR), AQP9 (up compared to CD4_NR), LAMP3 (up compared to CD4 PD_NR and HC_NR), PLK1 (down compared to HC_NR), and MPO (down compared to CD4 PD_NR).

### Enrichment of PD gene signature in CD4 and CD8 memory T cells

**Fig. 3:**
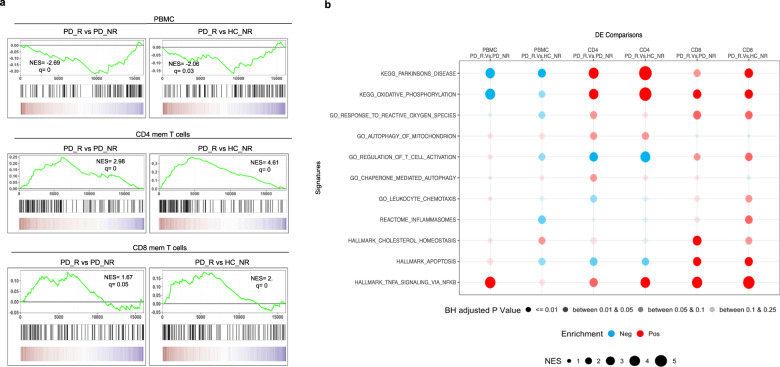
GSEA of the protein-coding transcriptome of PD_R vs. PD_NR and PD_R vs. HC_NR reveals enrichment of PD-associated gene signature in CD4 and CD8 memory T cells. **a** GSEA for the KEGG PD gene set. The *y*-axis of the plot shows the enrichment score (ES) for the gene set as the analysis moves down the ranked list of genes. The direction of the peak shows the degree to which the gene set is represented at the top or bottom of the ranked list of genes. The black bars on the *x*-axis show where the genes in the ranked list appear. The red portion at the bottom shows genes upregulated in PD_R and the blue portions represent the genes downregulated in PD_R (upregulated in HC_NR or PD_NR). q false discovery rate, NES normalized enrichment score. **b** Bubble plot demonstrating the enrichment status of several pathways previously reported to be implicated in PD. The red bubble indicates positive enrichment and the blue bubble indicates negative enrichment. The size of the bubble is directly proportional to the normalized enrichment score and the color shade of the bubble is proportional to the adjusted *p* value, where a darker bubble indicates higher significance than the lighter shade.

To further characterize the genes differentially expressed in PD_R, HC_NR, and PD_NR, we performed gene set enrichment analysis (GSEA)^22^. To check the enrichment of PD-associated gene signature in the differentially expressed genes between PD_R vs. HC_NR and PD_R vs. PD_NR, the DE genes were ranked and compared to an existing gene set “KEGG PARKINSONS DISEASE” that was downloaded from MSigDB in GMT format^23^. As shown in Fig. 3, a significant enrichment of PD-associated genes in PD_R was observed in CD4 and CD8 memory T cells. However, no such enrichment was observed in PBMCs (Fig. 3a).

We next examined the enrichment of several pathways implicated in PD, including oxidative phosphorylation^24^, oxidative stress^25–29^, macroautophagy and chaperone-mediated autophagy^30–34^, cholesterol signaling^35,36^, inflammation^6^, and TNF signaling^37^. Interestingly, chemotaxis, apoptosis, cholesterol biosynthesis, and inflammation were significantly enriched in CD8 memory T cells and oxidative stress, autophagy of mitochondria, and chaperone-mediated autophagy were enriched in CD4 memory T cells. Other pathways, such as oxidative phosphorylation and TNF signaling were enriched in both memory T cell subsets (Fig. 3b).

The results suggest that classifying the PD subjects based on their α-syn T cell reactivity and separately examining memory CD4 and CD8 T cell subsets can detect PD-associated gene signatures and identify PD-relevant pathways (Fig. 3a, b). It further suggests that peripheral memory T cell subsets might offer an opportunity to dissect the molecular mechanisms associated with PD pathogenesis. and is consistent with the notion that memory T cells may play a significant role in PD pathogenesis.

### Identification of cell surface and secreted protein targets

Because cell surface-expressed or secreted targets are amenable to modulation by monoclonal antibody therapy, we were interested in identifying which of the differentially expressed genes encode surface-expressed or secreted products that could be targeted in PD. We performed surfaceome and secretome analysis on the differentially expressed genes between PD_R vs. HC_NR and PD_R vs. PD_NR in all cell types. For surfaceome analysis, three databases of surface expressing targets^38–40^ were combined and a reference master list of targets that appeared in two out of three databases was generated that comprised of a total of 1168 targets. For secretome analysis, a reported human secretome database that comprised of 8575 targets was referred^41^. Combining surfaceome and secretome, we identified 133 and 76 targets that were either secretory and/or surface expressed in PD_R vs. PD_NR, and PD_R vs. HC_NR, respectively, in the CD4 memory T cell subset. We identified 140 and 100 targets in PD_R vs. PD_NR, and PD_R vs. HC_NR, respectively, in the CD8 memory T cell subset (Supplementary Table 2).

### Validation of potential genes of interest

We then selected specific DE genes for validation by flow cytometry-based on the availability of commercially available antibodies. Specifically, we validated one DE gene in each cell subset (CCR5 in PBMC; CX3CR1 in memory CD4 subset, and CCR1 in memory CD8 subset) at the protein level. The normalized expression count of the genes that were validated is represented in Supplementary Fig. 3A. The protein expression profile of the selected genes largely matched to the gene expression pattern observed by RNAseq analysis (Supplementary Fig. 3B). For example, PBMCs of HC_NR displayed significantly higher expression of CCR5 than PD_R, the CD4 memory subset of PD_NR had higher expression of CX3CR1 than PD_R, and the CD8 memory subset of PD_R had significantly higher expression of CCR1 than PD_NR and HC_NR. Similar trends were observed at the transcriptional and protein levels.

## Discussion

In this study, we show that memory T cells of PD subjects with detectable α-syn responses possess specific mRNA signatures. These signatures are associated with both known genes previously associated with neurological diseases and novel genes. The specific genes and pathways identified that show significant enrichment of transcriptomic signatures previously associated with PD include oxidative stress, oxidative phosphorylation, autophagy of mitochondria, chaperone-mediated autophagy, cholesterol metabolism, and inflammation. These molecular pathways and the associated genes are known to be dysregulated in PD and are widely thought to accelerate the progression of the disease. For instance, dysfunctional autophagic machinery leads to the accumulation of α-syn^42^ and defective mitochondria^43^ which in turn can lead to the formation of α-syn aggregates or impair energy metabolism and cause oxidative stress. Moreover, the accumulated and misfolded α-syn, a protein normally involved in the regulation of synaptic vesicle exocytosis^44^, causes degeneration of SNpc DA neurons, impairs synapse function^45–49^ and affects respiration, morphology, and turnover of mitochondria^50–57^, which may be related to display of mitochondrial-derived antigens in PD^58,59^. Additionally, cholesterol metabolism has also been linked to PD^60^ via a potential role in synaptogenesis. The interplay of implicated pathways suggests that a cascade of several molecular events takes place, resulting in progressive neurodegeneration.

We observed enrichment of reactive inflammasomes in CD8 memory T cell subset of PD responders, but not in their CD4 memory T cell subset, suggesting that PD-associated inflammatory signature is cell type-specific. We focused on the signatures associated with CD4 and CD8 memory T cells. The focus on T cells is prompted and supported by several reports that imply a T cell-associated inflammatory process^8,61^ within the PD prodromal phase and disease progression as well as in animal models^58^. Specific transcriptomic signatures associated with CD4 and CD8 memory T cell compartments have been described in several other pathologies^62–67^, including autoimmunity^68–70^, but here we report these types of signatures associated with memory T cells in neurodegenerative disease. A key element in our study was to focus on the transcriptional profile of specific purified memory CD4 and CD8 T cell subsets. Should this important aspect not have been considered, most of the differentially expressed genes and associated signatures would have been missed, as exemplified by the fact that very few differentially expressed genes were detected when whole PBMCs were considered.

As recently shown for monocytes, there can be a striking effect of sex on gene expression^71^. The DE genes detected in this study did not suggest sex-specific differences and there was an equal distribution of males and females in the PD-R and PD-NR cohort. Future studies with larger cohorts can provide further insights into the potential differential effects of sex on these signatures.

Transcriptional signatures associated with PD have been reported by several groups based on analysis of samples of neural origin that includes astrocytes, neurons, and brain tissue, including SN^72–76^. Here, we studied the signatures of T cells isolated from peripheral blood, rather than the CNS, because of the difficulty of accessing the CNS, and importantly, because of the lack of availability of sufficient numbers of T cells available to study in CNS fluids from PD donors and in particular from HC subjects^77^. While future studies might further investigate T cells isolated directly from the CNS, it is known that infiltrating T cells recirculate between the blood and the CNS^77,78^. To that end, we detected multiple differences in chemokine receptor expression between our PD_R group compared to PD_NR and/or HC_NR. This included reduced CCR5 in PD_R PBMC, as well as a reduction in CX3CR1 signal in PD_R memory CD4 T cells. Interestingly, CCR5 inhibitors have recently been shown to be therapeutic in a non-human primate model of PD^79^. As for CX3CR1, its potential role in PD is mainly thought to be mediated through microglia^80^; however, the receptor has been shown to define T cell memory populations^81^ which have implications in disease^82^. In terms of PD pathogenesis, the reduced amount of circulating CCR5 or CX3CR1 expressing T cells in PD individuals might indicate an increased accumulation of those cells in the brain parenchyma where they could contribute to local inflammation.

Some of the DE genes found in PBMCs and T cells are implicated in PD pathogenesis. This includes leucine-rich repeat kinase 2 (LRRK2), which is one of the two most common genes associated with familial PD and is also associated with sporadic PD. It has been noted that LRRK2 is far more highly expressed in immune cells than neurons, and is also linked to Crohn’s disease, an inflammatory bowel disorder, a class of disorders associated with PD^83^. LRRK2 expression in PBMCs may be related to the regulation of peripheral Type 2 interferon response that leads to dopamine neurodegeneration^84^, and its overall expression in T cells and other immune cells can be increased by interferon. In our results, LRRK2 transcript is decreased in PD to levels that are 33% the amount in HC. It could be that some PD-associated gene variations (whether as risk factors or familial mutations) influence not only CNS pathology but have a role in the inflammatory T cell response observed in PD. In terms of clinical backgrounds of the PD cohort e.g., familial, idiopathic, or mixed PD—while atypical parkinsonism and other neurological disorders were used as an exclusion criterion for enrollment, family history of PD was not. Self-reported family history of PD was captured in 41/56 of our PD subjects. Of the 41, 12 individuals reported some degree of PD in the family. Interestingly, the synuclein reactivity among those 12 individuals was 42% (5/12). Future studies examining the specific T cell response in PD populations with known familial mutations or risk alleles could better elucidate the potential link between the two.

Additional genes associated with mechanisms implicated in PD pathogenesis are also differentially expressed in T cells from PD_R subjects, including septin 5^85^, the GDNF receptor^86^, monoamine oxidase S, aquaporin^87^, LAMP3^88^ which has also been associated with a REM sleep disorder (a risk factor for PD^89^), polo-like kinase 1^90^, and myeloperoxidase^91^. Most of these genes have been found previously to be expressed in neurons, but here we show DE of these genes in peripheral cells. Moreover, these and additional DE genes point to the possibility that initiating steps in some PD pathogenic pathways might occur in peripheral immune cells and contribute to multiple hits that lead to the loss of targeted neurons^92^.

Another key element in our study was a focus on the transcriptional profile of PD subjects that were classified based on their T cell responsiveness to α-syn, which were taken as a proxy for subjects undergoing an ongoing inflammatory autoimmune process. This was a determinant aspect, and if this important aspect not have been considered, most of the differentially expressed genes and associated signatures would have been missed. The classification of subjects based on T cell reactivity of α-syn might be further refined by considering additional antigens other than α-syn that might be also involved in PD pathogenesis^93–95^. It is important to note here however the potential non-canonical effect α-syn might have on T cells directly as it has historically been shown to be pathogenic within neurons^96,97^, as well as glia^98–100^.

Based on a recently published conceptual model to describe PD pathogenesis^101^, factors that contribute to neurodegeneration can be divided into three categories: triggers, facilitators, and aggravators. Our study design focused on diagnosed PD patients with established disease and is therefore likely addressing factors that contribute to disease spread (facilitators) and promote the neurodegenerative process (aggravators). Unfortunately, we did not have any information on any particular PD subjects’ disease progression or their years since the onset of symptoms. It would be a focus of a future study of ours to further correlate α-syn reactivity to more detailed clinical characteristics in individuals with PD. Additionally, studies designed to look at risk categories for PD such as REM sleep disorder cohorts might shed light on RNA signatures associated with disease triggers.

Our data identify specific genes that could be addressed by therapeutic and diagnostic interventions, including TFEB, PRKN, and LRRK2. In a diagnostic setting, detection of alterations in the expression of these genes could contribute to a molecularly-based diagnostic, while in the therapeutic setting, it is possible that early targeting of the same genes by inhibiting or activating their function could delay or terminate disease progression or prevent disease development during the prodromal phase. Supportive of this notion is consistent with the observation that anti-TNF treatment^102^ is associated with lower PD disease incidence.

## Methods

### Ethics statement

All participants provided written informed consent for participation in the study. Ethical approval was obtained from the Institutional review boards at La Jolla Institute for Immunology (LJI; Protocol Nos. VD-124 and VD-118), Columbia University Medical Center (CUMC; protocol number IRB-AAAQ9714 and AAAS1669), University of California San Diego (UCSD; protocol number 161224), Rush University Medical Center (RUMC; Office of Research Affairs No.16042107-IRB01) and the University of Alabama at Birmingham (UAB; protocol number IRB-300001297).

### Study subjects

For RNAseq, we recruited a total of 56 individuals diagnosed with PD (*n* = 36) and age-matched healthy subjects (*n* = 20) in this study. The subjects were recruited from multiple sites: 32 subjects from Columbia University Medical Center (CUMC) (PD *n* = 26 and HC *n* = 6), 10 subjects from La Jolla Institute for Immunology (LJI) (PD *n* = 4 and HC *n* = 6), 8 subjects from University of California San Diego (UCSD) (PD *n* = 4 and HC *n* = 4), 3 subjects from Rush University Medical Center (RUMC) (PD *n* = 1 and HC *n* = 2), 3 subjects from University of Alabama at Birmingham (UAB) (PD *n* = 1 and HC *n* = 2). For the validation cohort, we analyzed 30 subjects: 20 PD and 10 HC. The subjects were recruited from multiple sites: 10 subjects from Columbia University Medical Center (CUMC) (PD *n* = 10), 12 subjects from La Jolla Institute for Immunology (LJI) (PD *n* = 2 and HC *n* = 10), 8 subjects from University of Alabama at Birmingham (UAB) (PD *n* = 8). The characteristics of the enrolled subjects are detailed in Table 2.

**Table 2:** Characteristics of the subjects enrolled in the study.

	RNAseq cohort			Validation cohort		
	PD_R	PD_NR	HC_NR	PD_R	PD_NR	HC_NR
Total subjects enrolled	15	21	20	10	10	10
Median age (range), yr	70 (49–81)	66 (44–81)	67 (50–79)	67 (46–81)	65 (44–76)	52 (22–69)
Male, % (*n*)	73.3% (11)	80.9% (17)	20% (4)	70% (7)	70% (7)	50% (5)
Caucasian, % (*n*)	100% (15)	85.7% (18)	80.9% (17)	90% (9)	100% (10)	50% (5)
Median years since diagnosis, (range), yr	3 (0–12)	6 (0–16)	NA	5 (1–12)	11 (1–19)	NA
Median MoCA^a^ (range)	27 (9–30)	26 (23–30)	NA	28 (22–30)	28 (14–30)	NA
Median UPDRS part III^b^ (range)	18 (7–37)	20 (5–30)	NA	19 (14–25)	18 (11–52)	NA

^a^MoCA collected for *n* = 32 PD patients in the RNAseq cohort and *n* = 17 in the validation cohort.

^b^UPDRS part III was collected during the on-phase for *n* = 31 PD patients in the RNAseq cohort and =17 in the validation cohort.

The cohorts were recruited by the clinical core at LJI, by the Parkinson and Other Movement Disorder Center at UCSD, the clinical practice of the UAB Movement Disorders Clinic, and the Movement Disorders Clinic at the Department of Neurology at CUMC. PD patients were enrolled on the basis of the following criteria: moderate to advanced PD; 2 of rest tremor, rigidity, and/or bradykinesia, PD diagnosis at age 45–80, dopaminergic medication benefit, and ability to provide informed consent. The exclusion criteria were atypical parkinsonism or other neurological disorders, history of cancer within the past 3 years, autoimmune disease, and chronic immune-modulatory therapy. Age-matched HC was selected on the basis of age 45–85 and ability to provide written consent. Exclusion criteria were the same as for PD donors and in addition, we excluded self-reported genetic factors. The HC was not screened for prodromal symptoms. The PD patients recruited at RUMC, UAB, CUMC, and UCSD (i.e., not at LJI) all fulfilled the UK Parkinson’s Disease Society Brain Bank criteria for PD. Patients with 0 years since diagnosis describe patients that had donated within their first year of being diagnosed with Parkinson’s disease.

### Peptides

Peptides were commercially synthesized on a small scale (1 mg/ml) by A&A, LLC (San Diego). A total of 11 peptides of α-syn^7^ were synthesized as purified material (>95% by reverse phase HPLC) and then reconstituted in DMSO at a concentration of 40 mg/ml. The individual peptides were then pooled, lyophilized, and reconstituted at a concentration of 3.6 mg/ml. The peptide pools were tested at a final concentration of 5 µg/ml.

### PBMC isolation

Venous blood was collected in heparin or EDTA-containing blood bags and PBMCs were isolated by density gradient centrifugation using Ficoll-Paque Plus (GE #17144003). Whole blood was first spun at 1850 rpm for 15 min with brakes off to remove plasma. The plasma-depleted blood was then diluted with RPMI and 35 ml of blood was gently layered on tubes containing 15 ml Ficoll–Paque plus. The tubes were then centrifuged at 1850 rpm for 25 min with brakes off. The cells at the interface were collected, washed with RPMI, counted, and cryopreserved in 90% v/v fetal bovine serum (FBS) and 10% v/v DMSO and stored in liquid nitrogen. The detailed protocol for PBMC isolation can be found at protocols.io (10.17504/protocols.io.bw2ipgce).

### Cell sorting and flow cytometry

The cryopreserved PBMC were thawed and revived in prewarmed RPMI media supplemented with 5% human serum (Gemini Bio-Products, West Sacramento, CA), 1% Glutamax (Gibco, Waltham, MA), 1% penicillin/streptomycin (Omega Scientific, Tarzana, CA), and 50 U/ml Benzonase (Millipore Sigma, Burlington, MA). The cells were then counted using a hemocytometer, washed with PBS, and prepared for staining. The cells at a density of 1 million were first incubated at 4 °C with 10% FBS for 10 min for blocking and then stained with a mixture of the following antibodies: APCef780 conjugated anti-CD4 (clone RPA-T4, eBiosciences, RRID:AB_1272044), AF700 conjugated anti-CD3 (clone UCHT1, BD Pharmigen, RRID:AB_10597906), BV650 conjugated anti-CD8a (clone RPA-T8, Biolegend, RRID:AB_11125174), PECy7 conjugated anti-CD19 (clone HIB19, TONBO, RRID:AB_2621841), APC conjugated anti-CD14 (clone 61D3, TONBO, RRID:AB_2621560), PerCPCy5.5 conjugated anti-CCR7 (clone G043H7, Biolegend, RRID:AB_10916121), PE-conjugated anti-CD56 (eBiosciences, RRID:AB_10598200), FITC conjugated anti-CD25 (clone M-A251, BD Pharmigen, RRID:AB_395825), eF450 conjugated anti-CD45RA (clone HI100, eBiosciences, RRID:AB_1272059) and eF506 live dead aqua dye (eBiosciences, 65-0866-1) for 30 min at 4 °C. Cells were then washed twice and resuspended in 100 µl PBS for flow cytometric analysis and sorting. The cells were sorted using BD FACSAria- (BD Biosciences) into ice-cold Trizol LS reagent (Thermo Fisher Scientific). The protocol can be found at protocols.io (10.17504/protocols.io.bwu9pez6). For the validation experiments, cells were processed in a similar manner as mentioned above and then stained with the following antibodies, AF700 conjugated anti-CD3 (clone UCHT1, BD Pharmigen, RRID:AB_10597906), BV650 conjugated anti-CD8a (clone RPA-T8, Biolegend, RRID:AB_11125174), eF450 conjugated anti-CD45RA (clone HI100, eBiosciences, RRID:AB_1272059), PerCPCy5.5 conjugated anti-CCR7 (clone G043H7, Biolegend, RRID:AB_10916121), BV786 conjugated anti-CD4 (clone SK3, BD Biosciences, RRID:AB_2738462), FITC conjugated anti-CD26 (clone BA5b, BioLegend, RRID:AB_314288), PECy7 conjugated anti-CCR1 (clone 5F10B29, BioLegend, RRID:AB_2734400), PE conjugated anti-CX3CR1 (clone 2A9-1, BioLegend, RRID:AB_1595456), BV605 conjugated anti-CCR5 (clone 2D7/CCR5, BD Biosciences, RRID:AB_2738167), PE-CF594 conjugated anti-CTLA-4 (clone BNI3, BD Biosciences, RRID:AB_2737761), APC-Cy7 conjugated anti-CD36 (clone 5-271, BioLegend, RRID:AB_2072512), and eF506 live dead aqua dye (eBiosciences, 65-0866-1) for 30 min at 4 °C. For the CTLA-4 staining, surface-stained cells were then permeabilized via 0.5% saponin buffer (Millipore Sigma, Burlington, MA) for 5 min at room temperature. CTLA-4 antibody was then incubated for 30 min at room temperature and afterwards cells were washed twice and resuspended in 100 µl PBS for flow cytometric analysis. Cells were acquired on a BD LSRFortessa (BD Biosciences). FCS files for cell surface and validation experiments were deposited to the open-access data depository (10.5281/zenodo.5523274 and 10.5281/zenodo.5248631, respectively).

### Fluorospot assay

PBMCs were thawed and stimulated for two weeks in vitro with α-syn pools. PHA was used as a control. Cells were fed with 10 U/ml recombinant IL-2 at an interval of 4 days. After 2 weeks of culture, T cell responses to α-syn were measured by IFNγ, IL-5, and IL-10 Fluorospot assay. Plates (Mabtech, Nacka Strand, Sweden) were coated overnight at 4 °C with an antibody mixture of mouse anti-human IFNγ (clone 1-D1K, RRID:AB_907283), mouse anti-human IL-5 (clone TRFK5, RRID:AB_907349), and mouse anti-human IL-10 (clone 9D7, RRID:AB_907307). Briefly, 100,000 cells were plated in each well of the pre-coated Immobilon-FL PVDF 96-well plates (Mabtech), stimulated with the respective antigen at the respective concentration of 5 μg/ml and incubated at 37 °C in a humidified CO_2_ incubator for 20–24 h. Cells stimulated with α-syn were also stimulated with 10 μg/ml PHA that served as a positive control. In order to assess nonspecific cytokine production, cells were also stimulated with DMSO at the corresponding concentration present in the peptide pools. All conditions were tested in triplicates. After incubation, cells were removed, plates were washed six times with 200 μl PBS/0.05% Tween 20 using an automated plate washer. After washing, 100 μl of an antibody mixture containing IFNγ (7-B6-1-FS-BAM), IL-5 (5A10-WASP), and IL-10 (12G8-biotin) prepared in PBS with 0.1% bovine serum albumin was added to each well, and plates were incubated for 2 h at room temperature. The plates were again washed six times as described above and incubated with diluted fluorophores (anti-BAM-490, anti-WASP-640, and SA-550—RRID:AB_907273, RRID:AB_907353, RRID:AB_907309) for 1 h at room temperature. After incubation, the plates were again washed as described above and incubated with a fluorescence enhancer for 15 min. Finally, the plates were blotted dry and spots were counted by computer-assisted image analysis (AID iSpot, AID Diagnostica GMBH, Strassberg, Germany). The responses were considered positive if they met all three criteria (i) the net spot forming cells per 10^6^ PBMC were ≥100 (ii) the stimulation index ≥ 2, and (iii) *p* ≤ 0.05 by Student’s *t* test or Poisson distribution test. Total fluorospot values according to donor groups were deposited to the open-access data depository (10.5281/zenodo.5703708). The full protocol is on protocols.io (10.17504/protocols.io.bpspmndn and 10.17504/protocols.io.bphjmj4n).

### Smart-seq

PBMC, CD4, and CD8 memory T cells of PD and HC subjects were sorted and total RNA from ~50,000 cells was extracted on a Qiacube using a miRNA easy kit (Qiagen, RRID:SCR_020419) and quantified using a bioanalyzer. Total RNA was amplified according to Smart Seq protocol^10^. cDNA was purified using AMPure XP beads. cDNA was used to prepare a standard barcoded sequencing library (Illumina). Samples were sequenced using an Illumina HiSeq2500 to obtain 50-bp single-end reads. Samples that failed to be sequenced due to limited sample availability or failed the quality control were eliminated from further sequencing and analysis. The full protocol can be found at protocols.io (10.17504/protocols.io.bxr6pm9e).

### RNA-seq analysis

The reads that passed Illumina filters were further filtered for reads aligning to tRNA, rRNA, adapter sequences, and spike-in controls. These reads were then aligned to the GRCh38 reference genome and Gencode v27 annotations using STAR: v2.6.1^103^. DUST scores were calculated with t, RRID:SCR_015687)^104^, and low-complexity reads (DUST > 4) were removed from the BAM files. The alignment results were parsed via the SAMtools (RRID:SCR_005611)^105^ to generate SAM files. Read counts to each genomic feature were obtained with featureCounts(v1.6.5, RRID:SCR_012919)^106^ with default options. After removing absent features (zero counts in all samples), the raw counts were then imported to R/Bioconductor package DESeq2 (v 1.24.0, RRID:SCR_015687)^107^ to identify differentially expressed genes among samples. Known batch conditions cohort and mapping run id were used in the design formula to correct for unwanted variation in the data. P-values for differential expression were calculated using the Wald test for differences between the base means of two conditions. These P-values are then adjusted for multiple test correction using the Benjamini Hochberg algorithm^108^. We considered genes differentially expressed between two groups of samples when the DESeq2 analysis resulted in an adjusted *p*-value of <0.05 and the difference in gene expression was 1.5-fold. The RNAseq data have been submitted to the Gene Expression Omnibus under accession number GSE174473.

### Gene set enrichment analysis

GSEA was done using the “GseaPreranked” method with “classic” scoring scheme and other default settings. The geneset KEGG PARKINSONS DISEASE was downloaded from MSigDB in GMT format (https://www.gseamsigdb.org/gsea/msigdb/cards/KEGG_PARKINSONS_DISEASE). Rank files for the DE comparisons of interest were generated by assigning a rank of −log10(*p* Value) to protein coding genes with log2FoldChange greater than zero and log10(*p* Value) to genes with log_2_ FoldChange less than zero. The GSEA figures were generated using ggplot2 package in R (RRID:SCR_014601) with gene ranks as the *x*-axis and enrichment score as the *y*-axis. The heatmap bar was generated using ggplot with genes ordered by their rank on *x*-axis and 1 as *y*-axis. Log_2_FoldChange values were used as the aes color option. scale_colour_gradient2 function was used with a midpoint=0 and other default options.

### Reporting summary

Further information on research design is available in the Nature Research Reporting Summary linked to this article.

## Supplementary information

Supplemental material

Reporting Summary Checklist

Dataset 1 (Supplementary table 1)

## Data Availability

Data generated or analyzed during this study are included in this article and its Supplementary Information. The datasets generated during and/or analyzed during the current study are available in the Gene Expression Omnibus under accession number GSE174473. Other datasets are available through the Zenodo data depository FCS: 10.5281/zenodo.5523274 and 10.5281/zenodo.5248631. Fluorospot: 10.5281/zenodo.5703708. Experimental protocols are available on protocols.io.

## References

[1] Fahn S, Sulzer D (2004). Neurodegeneration and neuroprotection in Parkinson disease. NeuroRx.

[2] Spillantini MG (1997). Alpha-synuclein in Lewy bodies. Nature.

[3] Archibald N, Miller N, Rochester L (2013). Neurorehabilitation in Parkinson disease. Handb. Clin. Neurol..

[4] Marras C (2018). Prevalence of Parkinson’s disease across North America. NPJ Parkinsons Dis..

[5] McGeer PL, Itagaki S, Boyes BE, McGeer EG (1988). Reactive microglia are positive for HLA-DR in the substantia nigra of Parkinson’s and Alzheimer’s disease brains. Neurology.

[6] Stojkovska I, Wagner BM, Morrison BE (2015). Parkinson’s disease and enhanced inflammatory response. Exp. Biol. Med..

[7] Sulzer D (2017). T cells from patients with Parkinson’s disease recognize alpha-synuclein peptides. Nature.

[8] Lindestam Arlehamn CS (2020). alpha-Synuclein-specific T cell reactivity is associated with preclinical and early Parkinson’s disease. Nat. Commun..

[9] Postuma RB, Berg D (2016). Advances in markers of prodromal Parkinson disease. Nat. Rev. Neurol..

[10] Picelli S (2014). Full-length RNA-seq from single cells using Smart-seq2. Nat. Protoc..

[11] Dachsel JC, Farrer MJ (2010). LRRK2 and Parkinson disease. Arch. Neurol..

[12] Dawson TM, Dawson VL (2010). The role of parkin in familial and sporadic Parkinson’s disease. Mov. Disord..

[13] Di Maio, R. et al. LRRK2 activation in idiopathic Parkinson’s disease. *Sci. Transl. Med.*10.1126/scitranslmed.aar5429 (2018). 10.1126/scitranslmed.aar5429 PMC6344941 30045977

[14] Li JQ, Tan L, Yu JT (2014). The role of the LRRK2 gene in Parkinsonism. Mol. Neurodegener..

[15] Mata IF, Wedemeyer WJ, Farrer MJ, Taylor JP, Gallo KA (2006). LRRK2 in Parkinson’s disease: protein domains and functional insights. Trends Neurosci..

[16] Rui Q, Ni H, Li D, Gao R, Chen G (2018). The role of LRRK2 in neurodegeneration of Parkinson disease. Curr. Neuropharmacol..

[17] von Coelln R, Dawson VL, Dawson TM (2004). Parkin-associated Parkinson’s disease. Cell Tissue Res..

[18] Settembre C (2011). TFEB links autophagy to lysosomal biogenesis. Science.

[19] Decressac M, Bjorklund A (2013). TFEB: pathogenic role and therapeutic target in Parkinson disease. Autophagy.

[20] Torra A (2018). Overexpression of TFEB drives a pleiotropic neurotrophic effect and prevents Parkinson’s disease-related neurodegeneration. Mol. Ther..

[21] Zhuang XX (2020). Pharmacological enhancement of TFEB-mediated autophagy alleviated neuronal death in oxidative stress-induced Parkinson’s disease models. Cell Death Dis..

[22] Subramanian A (2005). Gene set enrichment analysis: a knowledge-based approach for interpreting genome-wide expression profiles. Proc. Natl Acad. Sci. USA.

[23] Liberzon A (2011). Molecular signatures database (MSigDB) 3.0. Bioinformatics.

[24] Shoffner JM, Watts RL, Juncos JL, Torroni A, Wallace DC (1991). Mitochondrial oxidative phosphorylation defects in Parkinson’s disease. Ann. Neurol..

[25] Blesa J, Trigo-Damas I, Quiroga-Varela A, Jackson-Lewis VR (2015). Oxidative stress and Parkinson’s disease. Front. Neuroanat..

[26] Dias V, Junn E, Mouradian MM (2013). The role of oxidative stress in Parkinson’s disease. J. Parkinsons Dis..

[27] Hemmati-Dinarvand M (2019). Oxidative stress and Parkinson’s disease: conflict of oxidant-antioxidant systems. Neurosci. Lett..

[28] Hwang O (2013). Role of oxidative stress in Parkinson’s disease. Exp. Neurobiol..

[29] Jenner P (2003). Oxidative stress in Parkinson’s disease. Ann. Neurol..

[30] Hou X, Watzlawik JO, Fiesel FC, Springer W (2020). Autophagy in Parkinson’s disease. J. Mol. Biol..

[31] Lynch-Day MA, Mao K, Wang K, Zhao M, Klionsky DJ (2012). The role of autophagy in Parkinson’s disease. Cold Spring Harb. Perspect. Med..

[32] Moors TE (2017). Therapeutic potential of autophagy-enhancing agents in Parkinson’s disease. Mol. Neurodegener..

[33] Wang B, Abraham N, Gao G, Yang Q (2016). Dysregulation of autophagy and mitochondrial function in Parkinson’s disease. Transl. Neurodegener..

[34] Zhang L, Dong Y, Xu X, Xu Z (2012). The role of autophagy in Parkinson’s disease. Neural Regen. Res..

[35] Jin U, Park SJ, Park SM (2019). Cholesterol metabolism in the brain and its association with Parkinson’s disease. Exp. Neurobiol..

[36] Vance JE (2012). Dysregulation of cholesterol balance in the brain: contribution to neurodegenerative diseases. Dis. Model. Mech..

[37] Leal MC, Casabona JC, Puntel M, Pitossi FJ (2013). Interleukin-1beta and tumor necrosis factor-alpha: reliable targets for protective therapies in Parkinson’s disease?. Front. Cell Neurosci..

[38] Ashburner M (2000). Gene ontology: tool for the unification of biology. The Gene Ontology Consortium. Nat. Genet..

[39] Bausch-Fluck D (2018). The in silico human surfaceome. Proc. Natl Acad. Sci. USA.

[40] Bausch-Fluck D (2015). A mass spectrometric-derived cell surface protein atlas. PLoS ONE.

[41] Vathipadiekal V (2015). Creation of a human secretome: a novel composite library of human secreted proteins: validation using ovarian cancer gene expression data and a virtual secretome array. Clin. Cancer Res..

[42] Martinez-Vicente M (2008). Dopamine-modified alpha-synuclein blocks chaperone-mediated autophagy. J. Clin. Invest..

[43] Lee J, Giordano S, Zhang J (2012). Autophagy, mitochondria and oxidative stress: cross-talk and redox signalling. Biochem. J..

[44] Somayaji M (2020). A dual role for alpha-synuclein in facilitation and depression of dopamine release from substantia nigra neurons in vivo. Proc. Natl Acad. Sci. USA.

[45] Chung CY, Koprich JB, Siddiqi H, Isacson O (2009). Dynamic changes in presynaptic and axonal transport proteins combined with striatal neuroinflammation precede dopaminergic neuronal loss in a rat model of AAV alpha-synucleinopathy. J. Neurosci..

[46] Ihara M (2007). Sept4, a component of presynaptic scaffold and Lewy bodies, is required for the suppression of alpha-synuclein neurotoxicity. Neuron.

[47] Kahle PJ (2000). Subcellular localization of wild-type and Parkinson’s disease-associated mutant alpha-synuclein in human and transgenic mouse brain. J. Neurosci..

[48] Yavich L, Jakala P, Tanila H (2006). Abnormal compartmentalization of norepinephrine in mouse dentate gyrus in alpha-synuclein knockout and A30P transgenic mice. J. Neurochem..

[49] Sulzer D, Edwards RH (2019). The physiological role of alpha-synuclein and its relationship to Parkinson’s Disease. J. Neurochem..

[50] Chinta SJ, Mallajosyula JK, Rane A, Andersen JK (2010). Mitochondrial alpha-synuclein accumulation impairs complex I function in dopaminergic neurons and results in increased mitophagy in vivo. Neurosci. Lett..

[51] Choubey V (2011). Mutant A53T alpha-synuclein induces neuronal death by increasing mitochondrial autophagy. J. Biol. Chem..

[52] Cole NB, Dieuliis D, Leo P, Mitchell DC, Nussbaum RL (2008). Mitochondrial translocation of alpha-synuclein is promoted by intracellular acidification. Exp. Cell Res..

[53] Devi L, Raghavendran V, Prabhu BM, Avadhani NG, Anandatheerthavarada HK (2008). Mitochondrial import and accumulation of alpha-synuclein impair complex I in human dopaminergic neuronal cultures and Parkinson disease brain. J. Biol. Chem..

[54] Li WW (2007). Localization of alpha-synuclein to mitochondria within midbrain of mice. Neuroreport.

[55] Martin LJ (2006). Parkinson’s disease alpha-synuclein transgenic mice develop neuronal mitochondrial degeneration and cell death. J. Neurosci..

[56] Parihar MS, Parihar A, Fujita M, Hashimoto M, Ghafourifar P (2008). Mitochondrial association of alpha-synuclein causes oxidative stress. Cell Mol. Life Sci..

[57] Parihar MS, Parihar A, Fujita M, Hashimoto M, Ghafourifar P (2009). Alpha-synuclein overexpression and aggregation exacerbates impairment of mitochondrial functions by augmenting oxidative stress in human neuroblastoma cells. Int. J. Biochem. Cell Biol..

[58] Matheoud D (2019). Intestinal infection triggers Parkinson’s disease-like symptoms in Pink1(-/-) mice. Nature.

[59] McLelland GL, Soubannier V, Chen CX, McBride HM, Fon EA (2014). Parkin and PINK1 function in a vesicular trafficking pathway regulating mitochondrial quality control. EMBO J..

[60] Huang X (2019). Brain cholesterol metabolism and Parkinson’s disease. Mov. Disord..

[61] Seo J (2020). Chronic infiltration of T lymphocytes into the brain in a non-human primate model of Parkinson’s disease. Neuroscience.

[62] Burel JG (2018). Transcriptomic analysis of CD4(+) T cells reveals novel immune signatures of latent tuberculosis. J. Immunol..

[63] Grifoni A (2018). Cutting edge: transcriptional profiling reveals multifunctional and cytotoxic antiviral responses of zika virus-specific CD8(+) T cells. J. Immunol..

[64] Hyrcza MD (2007). Distinct transcriptional profiles in ex vivo CD4+ and CD8+ T cells are established early in human immunodeficiency virus type 1 infection and are characterized by a chronic interferon response as well as extensive transcriptional changes in CD8+ T cells. J. Virol..

[65] Tian Y (2019). Dengue-specific CD8+ T cell subsets display specialized transcriptomic and TCR profiles. J. Clin. Invest..

[66] Tian Y (2019). Molecular signatures of dengue virus-specific IL-10/IFN-gamma co-producing CD4 T cells and their association with dengue disease. Cell Rep..

[67] Patil, V. S. et al. Precursors of human CD4(+) cytotoxic T lymphocytes identified by single-cell transcriptome analysis. *Sci. Immunol*. 10.1126/sciimmunol.aan8664 (2018). 10.1126/sciimmunol.aan8664 PMC5931334 29352091

[68] Hong X (2020). Single-cell RNA sequencing reveals the expansion of cytotoxic CD4(+) T lymphocytes and a landscape of immune cells in primary Sjogren’s syndrome. Front Immunol..

[69] Lyons PA (2010). Novel expression signatures identified by transcriptional analysis of separated leucocyte subsets in systemic lupus erythematosus and vasculitis. Ann. Rheum. Dis..

[70] McKinney EF (2010). A CD8+ T cell transcription signature predicts prognosis in autoimmune disease. Nat. Med..

[71] Carlisle SM (2021). Sex-based differences in the activation of peripheral blood monocytes in early Parkinson disease. NPJ Parkinsons Dis..

[72] Keo A (2020). Transcriptomic signatures of brain regional vulnerability to Parkinson’s disease. Commun. Biol..

[73] Lang C (2019). Single-cell sequencing of iPSC-dopamine neurons reconstructs disease progression and identifies HDAC4 as a regulator of Parkinson cell phenotypes. Cell Stem Cell.

[74] Sandor C (2017). Transcriptomic profiling of purified patient-derived dopamine neurons identifies convergent perturbations and therapeutics for Parkinson’s disease. Hum. Mol. Genet..

[75] Booth HDE (2019). RNA sequencing reveals MMP2 and TGFB1 downregulation in LRRK2 G2019S Parkinson’s iPSC-derived astrocytes. Neurobiol. Dis..

[76] Nido GS (2020). Common gene expression signatures in Parkinson’s disease are driven by changes in cell composition. Acta Neuropathol. Commun..

[77] Ransohoff RM, Kivisakk P, Kidd G (2003). Three or more routes for leukocyte migration into the central nervous system. Nat. Rev. Immunol..

[78] Shechter R, London A, Schwartz M (2013). Orchestrated leukocyte recruitment to immune-privileged sites: absolute barriers versus educational gates. Nat. Rev. Immunol..

[79] Mondal S (2019). Low-dose maraviroc, an antiretroviral drug, attenuates the infiltration of T cells into the central nervous system and protects the nigrostriatum in Hemiparkinsonian monkeys. J. Immunol..

[80] Angelopoulou E, Paudel YN, Shaikh MF, Piperi C (2020). Fractalkine (CX3CL1) signaling and neuroinflammation in Parkinson’s disease: potential clinical and therapeutic implications. Pharm. Res..

[81] Gerlach C (2016). The chemokine receptor CX3CR1 defines three antigen-experienced CD8 T cell subsets with distinct roles in immune surveillance and homeostasis. Immunity.

[82] Yamauchi, T. et al. CX3CR1-CD8+ T cells are critical in antitumor efficacy but functionally suppressed in the tumor microenvironment. *JCI Insight*10.1172/jci.insight.133920 (2020). 10.1172/jci.insight.133920 PMC7205436 32255766

[83] Herrick MK, Tansey MG (2021). Is LRRK2 the missing link between inflammatory bowel disease and Parkinson’s disease?. NPJ Parkinsons Dis..

[84] Kozina E (2018). Mutant LRRK2 mediates peripheral and central immune responses leading to neurodegeneration in vivo. Brain.

[85] Son JH (2005). Neurotoxicity and behavioral deficits associated with Septin 5 accumulation in dopaminergic neurons. J. Neurochem..

[86] Sandmark J (2018). Structure and biophysical characterization of the human full-length neurturin-GFRa2 complex: a role for heparan sulfate in signaling. J. Biol. Chem..

[87] Tamtaji OR, Behnam M, Pourattar MA, Jafarpour H, Asemi Z (2019). Aquaporin 4: a key player in Parkinson’s disease. J. Cell Physiol..

[88] Liu X (2011). Genome-wide association study identifies candidate genes for Parkinson’s disease in an Ashkenazi Jewish population. BMC Med. Genet..

[89] Mufti K (2021). Novel associations of BST1 and LAMP3 with REM sleep behavior disorder. Neurology.

[90] Mbefo MK (2010). Phosphorylation of synucleins by members of the Polo-like kinase family. J. Biol. Chem..

[91] Maki RA (2019). Human myeloperoxidase (hMPO) is expressed in neurons in the substantia nigra in Parkinson’s disease and in the hMPO-alpha-synuclein-A53T mouse model, correlating with increased nitration and aggregation of alpha-synuclein and exacerbation of motor impairment. Free Radic. Biol. Med..

[92] Raj T (2014). Polarization of the effects of autoimmune and neurodegenerative risk alleles in leukocytes. Science.

[93] Latorre D (2018). T cells in patients with narcolepsy target self-antigens of hypocretin neurons. Nature.

[94] Lindestam Arlehamn CS (2019). Widespread tau-specific CD4 T cell reactivity in the general population. J. Immunol..

[95] Lodygin D (2019). beta-Synuclein-reactive T cells induce autoimmune CNS grey matter degeneration. Nature.

[96] Luk KC (2012). Pathological alpha-synuclein transmission initiates Parkinson-like neurodegeneration in nontransgenic mice. Science.

[97] Stefanis L (2012). alpha-Synuclein in Parkinson’s disease. Cold Spring Harb. Perspect. Med..

[98] Fellner L (2013). Toll-like receptor 4 is required for alpha-synuclein dependent activation of microglia and astroglia. Glia.

[99] Lee HJ (2010). Direct transfer of alpha-synuclein from neuron to astroglia causes inflammatory responses in synucleinopathies. J. Biol. Chem..

[100] Scheiblich H (2021). Microglia jointly degrade fibrillar alpha-synuclein cargo by distribution through tunneling nanotubes. Cell.

[101] Johnson ME, Stecher B, Labrie V, Brundin L, Brundin P (2019). Triggers, facilitators, and aggravators: redefining Parkinson’s disease pathogenesis. Trends Neurosci..

[102] Peter I (2018). Anti-tumor necrosis factor therapy and incidence of Parkinson disease among patients with inflammatory bowel disease. JAMA Neurol..

[103] Dobin A (2013). STAR: ultrafast universal RNA-seq aligner. Bioinformatics.

[104] Schmieder R, Edwards R (2011). Quality control and preprocessing of metagenomic datasets. Bioinformatics.

[105] Li H (2009). The Sequence Alignment/Map format and SAMtools. Bioinformatics.

[106] Liao Y, Smyth GK, Shi W (2014). featureCounts: an efficient general purpose program for assigning sequence reads to genomic features. Bioinformatics.

[107] Love MI, Huber W, Anders S (2014). Moderated estimation of fold change and dispersion for RNA-seq data with DESeq2. Genome Biol..

[108] Benjamini Y, Hochberg Y (1995). Controlling the false discovery rate: a practical and powerful approach to multiple testing. J. R. Stat. Soc. Ser. B.

