# Supplementary material for: Transcriptional analysis of peripheral memory T cells reveals Parkinson’s disease-specific gene signatures
Source: NPJ Parkinsons Dis. 2022 Mar 21;8:30. doi: 10.1038/s41531-022-00282-2 (PMC8938520; doi:10.1038/s41531-022-00282-2)
Supplement: Supplementary file 1 — Supplemental material [file 41531_2022_282_MOESM1_ESM.pdf]

Supplementary figures

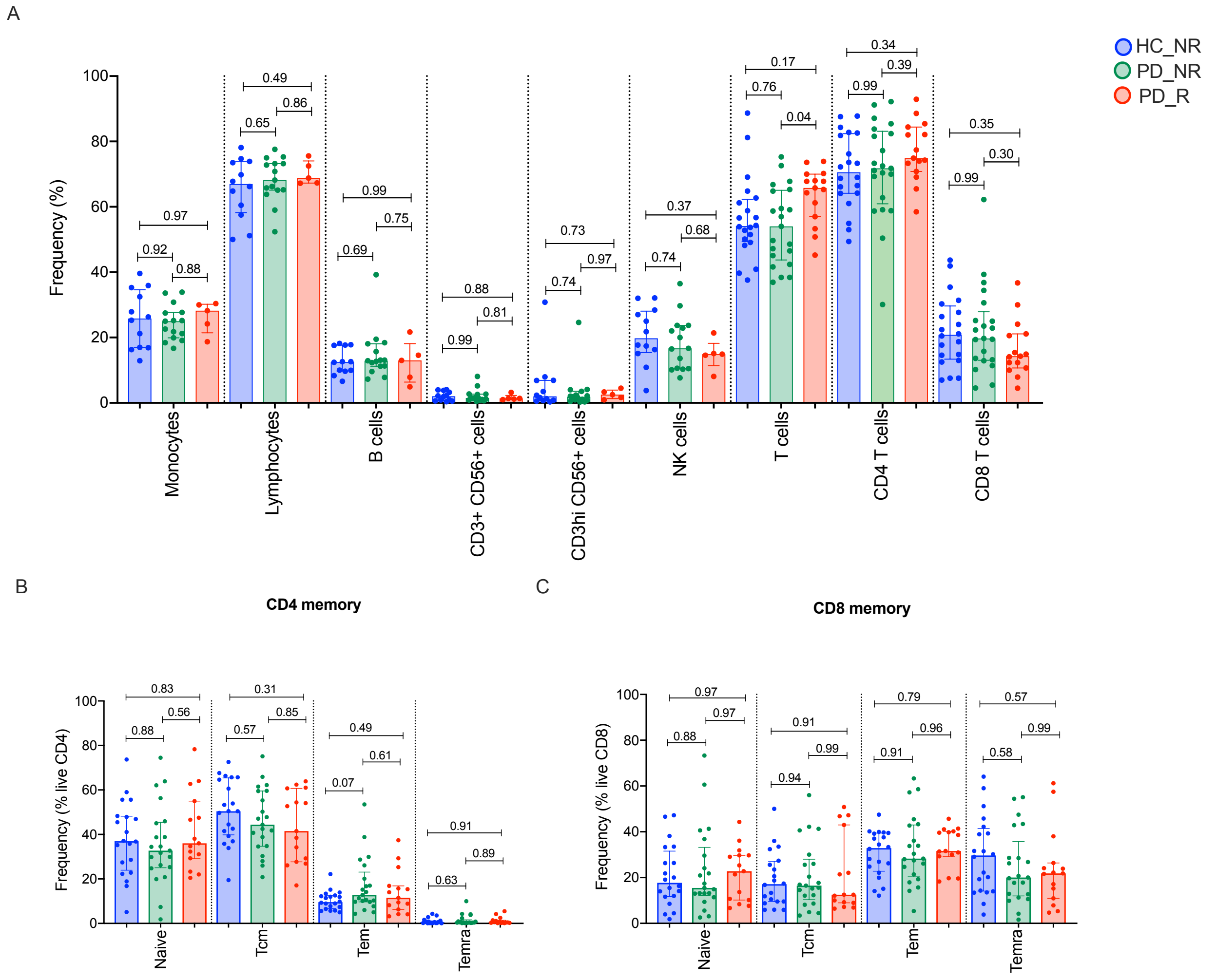

Supplementary Figure 1. Relative frequency of different cell subsets in HC_NR, PD_NR and PD_R. (A). Frequency of major PBMC subsets in HC_NR (blue bar and circles), PD_NR (green bars and circles) and PD_R (red bars and circles) (B) CD4 memory and (C) CD8 memory T cells were further evaluated for frequency of naïve, effector memory (T_{em}), central memory (T_{cm}) and T_{EMRA} populations. Each point represents a donor. Median ± interquartile range is displayed. Anova with multiple comparison Tukey correction.

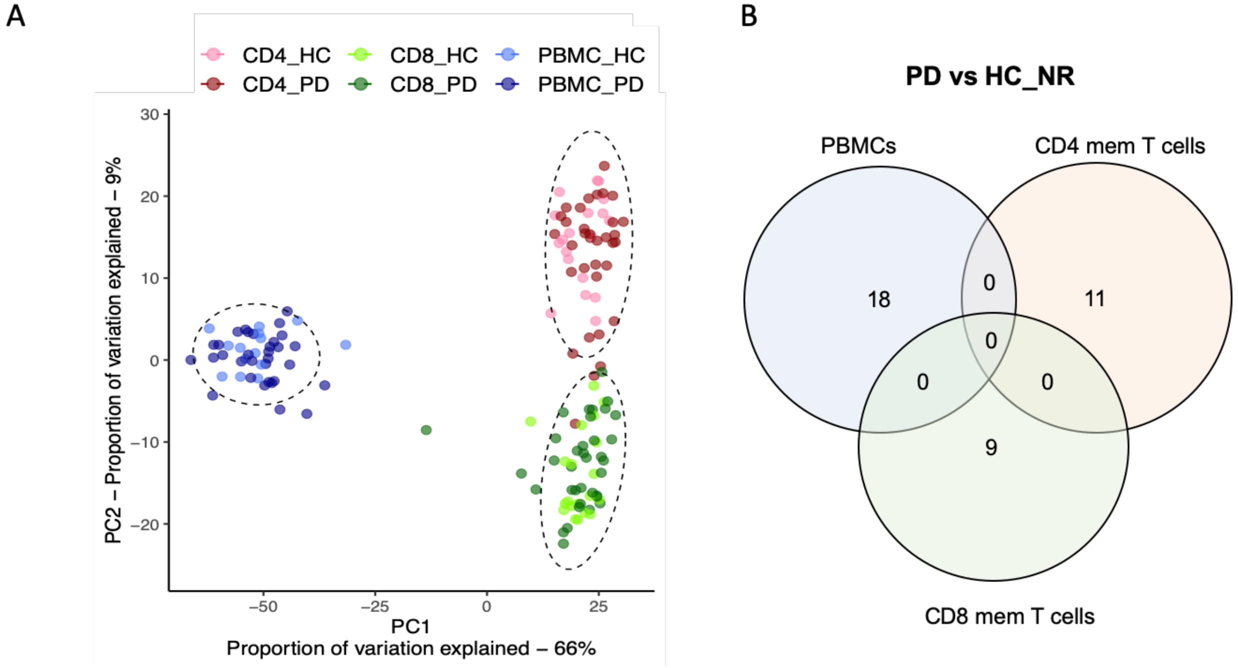

Supplementary Figure 2. Comparison of PD vs HC in PBMCs, CD4 and CD8 memory T cells (A) PCA plot demonstrating distinct profile of PBMCs, CD4 and CD8 memory T cells and no separation between PD and HC_NR in either cell type. (B) Venn diagram demonstrating the overlap between PBMC, CD4 and CD8 memory T cells.

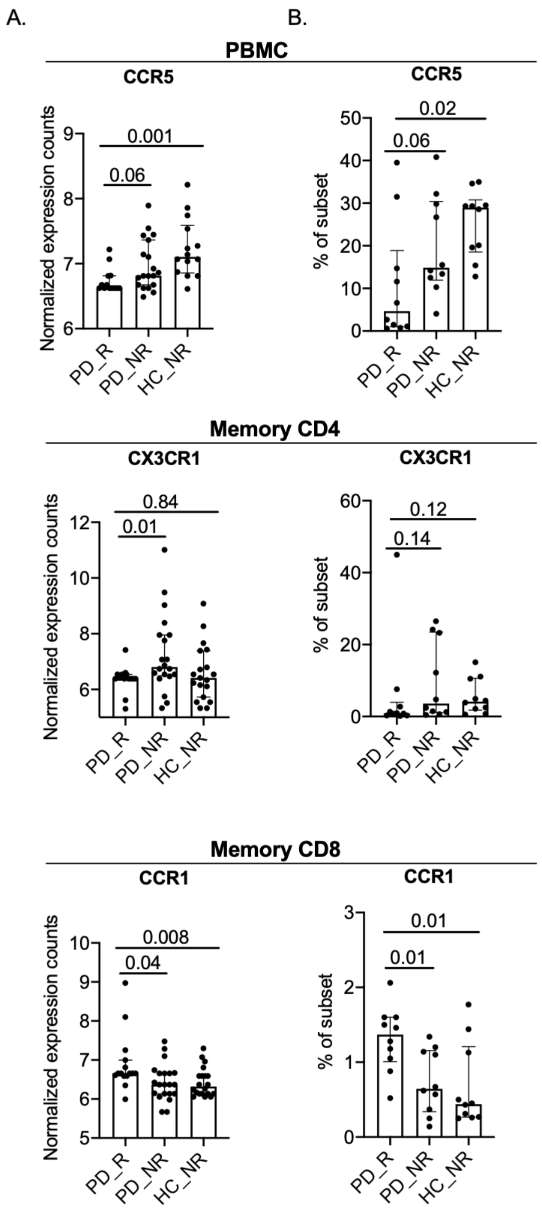

Supplementary Figure 3. Gene expression profile of specific DE genes in PBMC, CD4 memory and CD8 memory cell types. (A) Gene expression values of CCR5, CX3CR1, and CCR1 in counts normalized by sequencing depth calculated by DEseq2 package. (B) Protein expression as percent frequency of subset measured using flow cytometry. Median interquartile range is shown. Two-tailed Mann-Whitney test.

Supplementary Tables

Supplementary Table 1. List of DE genes for all cell types and comparisons

Provided as an excel file due to complexity.

Supplementary Table 2. Surface expressing and secretory targets in different comparisons: Genes in bold are both surface and secretory targets, unbolded genes are secretory, italicized genes are surface expressing targets. DE: Differentially expressed genes, SE: surface expressing/secretory target gene.

	**PBMC**			**CD4 memory T cells**			**CD8 memory T cells**		
**Comparison**	**PD vs HC**	**PD_R vs PD_NR**	**PD_R vs HC_NR**	**PD vs HC**	**PD_R vs PD_NR**	**PD_R vs HC_NR**	**PD vs HC**	**PD_R vs PD_NR**	**PD_R vs HC_NR**
**DE genes**	26	132	101	16	503	260	14	494	356
**Protein coding**	18	90	65	11	304	172	9	333	227
**SE genes**	9	39	25	4	133	76	3	140	100
**Down regulated Genes**	**DCHS1**<br>TMTC1<br>WDR5B<br>CACNA1F	POPDC2<br>GPR171<br>AATK<br>XPNPEP2<br>P2RY6<br>NAPSA<br>**SLC45A4**<br>PRPF40B<br>FAM173B<br>**SLC22A16**<br>DKK3<br>NUDT6<br>BIK<br>C1QC<br>PLCD3<br>MADCAM1<br>HAS1<br>**HFE**<br>ORMDL2<br>EMID1<br>C17orf80<br>STK32B<br>**SLC22A23**<br>**CCR5**<br>**CXCR1**<br>CLIP3<br>LRRC3<br>F2RL3<br>SLCO5A1<br>HIGD1A<br>*MRC1*<br>*F2RL3*	P2RY6<br>EPHX3<br>**NRP2**<br>**ACE**<br>**SLC22A16**<br>LRRC3<br>PRSS27<br>**HFE**<br>FFAR1<br>PRPF40B<br>**CCR5**<br>FUT2<br>SDCBP2<br>TMTC1<br>**DCHS1**<br>CTRC<br>COL4A2	ZACN<br>GREM2<br>CYP2U1	CALHM2<br>MAMDC4<br>HS6ST1<br>PEX26<br>CDC42EP2<br>**CD36**<br>**CD8A**<br>MMP25<br>CISH<br>TNFAIP2<br>FGR<br>ACAN<br>ABCC3<br>RAB6B<br>TMEM201<br>IGFBP6<br>**CX3CR1**<br>F2RL2<br>AP2A1<br>FBLN2<br>DHCR24<br>KCNN3<br>**ITGB4**<br>SPINK4<br>**MFSD8**<br>ST6GALNAC6<br>TBC1D8<br>FCGBP<br>**CSF3R**<br>XPNPEP2<br>**SIGLEC7**<br>SRC<br>WIPI1<br>LAT2<br>PSKH1<br>RAB3D<br>CDA<br>**SELPLG**<br>COL16A1<br>TOM1L1	SYK<br>CDA<br>LAT2<br>KCNH4<br>DHCR24<br>**CCR3**<br>NPHP4<br>F2RL2<br>COL16A1<br>CD300LB<br>**MSLN**<br>AP2A1<br>RAB26<br>IGFBP6<br>RNF152<br>ACAN<br>CPB2<br>**SLC15A2**<br>CYTL1<br>ZACN<br>LRRK2<br>TREML1<br>WIPI1<br>SEMA3G<br>**CD36**<br>SLC7A8<br>VSIG4<br>ABCC3<br>IL22<br>TOM1L1<br>FXYD6<br>FFAR3<br>GBGT1<br>STRC<br>SLC35F3<br>SCARA3<br>MCOLN3<br>**LAMP3**<br>ZNF532<br>TRPM2	SEMA6C<br>*PODXL*	KDELR2<br>DMXL2<br>KCNQ4<br>CNIH2<br>**IL10RB**<br>TMEM203<br>BAIAP2L1<br>MMP17<br>TACR2<br>**TMEM179B**<br>VAMP4<br>AMACR<br>RNF5<br>HIST1H4I<br>EPS8L1<br>CDC42EP2<br>HCN2<br>ZNRF3<br>PRKD2<br>PAQR4<br>KDELR1	PRKD2<br>ACRBP<br>KCNQ4<br>CNIH2<br>CD320<br>GIPR<br>SLC25A42<br>UPF3A<br>MAOA<br>BAIAP2L1<br>SLC25A17<br>PAQR4<br>SYT6<br>C14orf132<br>PELP1<br>VAMP4<br>REG4<br>SEMA6C<br>EPS8L1<br>*PODXL*

					**HHLA2**<br>KCNH4<br>RAB1B<br>RNF152<br>CPB2<br>CD300LB<br>SGCA<br>FFAR3<br>PBXIP1<br>**LRFN2**<br>SLC7A8<br>TAP1<br>CHRNA10<br>**RET**<br>EXOC3L2<br>IL22<br>ST3GAL6<br>**SLC15A2**<br>GPR153<br>MS4A14<br>KIRREL2<br>LRRK2<br>SLC30A8<br>MPO<br>OLFML2B<br>LYPD4<br>**IGDCC4**<br>GML<br>FGFBP1	MTUS1<br>PDIA5<br>AIG1<br>INVS<br>TMEM97<br>H6PD<br>FAM19A2<br>CYP2S1<br>LEAP2<br>CASK<br>SARNP<br>**PTGIR**<br>**CSF3R**<br>SLC25A30<br>RAB6B<br>TMEM201<br>C2CD2<br>CHRNA10<br>NAPA			
**Upregulated genes**	CLEC4F<br>AP3B2<br>ITPRIPL1<br>EIF1AY<br>CYP2F1	TPR<br>EMILIN2<br>ZNRF3<br>SPOCD1<br>CTDSPL<br>EYS<br>GCNT7	PCBP2<br>CERK<br>**LPAR1**<br>EIF1AY<br>PEX3<br>PDPR<br>**CRIM1**<br>CYP2F1	SOX12	GYPE<br>RASD1<br>**CD180**<br>B3GNT8<br>STRC<br>SLC7A10<br>TOMM5<br>RNASEL<br>ADCK1<br>FUT11<br>INVS<br>**CSF2RB**<br>EPHX1<br>HIST1H4H<br>SLC25A19<br>CYP2S1<br>TRPM2<br>MAN1C1	AQP9<br>ECE2<br>INSL4<br>TOMM5<br>SERPINH1<br>HIST1H4H<br>GDF11<br>ABCD2<br>CRB3<br>**CELSR2**<br>MPHOSPH9<br>**TNFRSF11A**<br>GALNT1<br>APOL1<br>DCST1<br>BAIAP2<br>SOX12	**GPM6B**	MSRB3<br>LTC4S<br>METTL7A<br>OVGP1<br>MAPK15<br>**SLC19A1**<br>MTX2<br>COL1A1<br>MBOAT2<br>SORD<br>HSD17B7<br>SLC4A8<br>**CDH5**<br>GNG12<br>**ANO6**<br>MAMLD1<br>C8G<br>ANKRD44	TMPRSS2<br>**GFRA2**<br>MFSD6L<br>LRRK2<br>SRC<br>RGMB<br>B4GALNT1<br>LRRC3<br>OVGP1<br>**CCR1**<br>**SLC19A1**<br>S100A8<br>G0S2<br>GNG12<br>SH2B2<br>FPR1<br>**PLXNA4**<br>LHFPL2

					PEX11G<br>NDUFC1<br>**CELSR2**<br>CREB3L4<br>MEGF6<br>MPP5<br>TMEM97<br>ECE2<br>GCNT2<br>SVIL<br>SARNP<br>PDIA5<br>**ADAM22**<br>ALS2<br>MGP<br>H6PD<br>PIGK<br>DENND1A<br>BCL2<br>CHMP5<br>CRB3<br>KCNQ1<br>**FAM171A1**<br>ZNF532<br>MTUS1<br>ISM1<br>DYNC2H1<br>NLRP2<br>AP2A2<br>SERPINH1<br>DCST1<br>APOL1<br>AIG1<br>**LAMP3**<br>ADCK5<br>**IL6R**<br>CASK<br>MPHOSPH9<br>**ALCAM**<br>FAM19A2<br>ABCD2<br>PXMP2<br>CNKSR2<br>GDF11<br>BAIAP2<br>LEAP2			PLEKHB1<br>TSSK4<br>**CALCRL**<br>MGLL<br>MFSD6L<br>QPCT<br>NDFIP2<br>MS4A6A<br>MARCO<br>CLCN1<br>SEMA3B<br>C2CD2<br>GPRIN1<br>CLN6<br>PLXDC2<br>KCNH3<br>APOO<br>ZNF599<br>B3GALT2<br>FBXO2<br>GLT1D1<br>LAPTM4B<br>**CTLA4**<br>**CCR1**<br>**CXADR**<br>FITM2<br>RASGRF2<br>MAP2K6<br>DRD3<br>S100A8<br>TPCN1<br>BCL7A<br>IL17C<br>ZDHHC14<br>LAT2<br>KIAA0319L<br>**NOTCH4**<br>**NTSR1**<br>**GFRA2**<br>HMOX1<br>RALGPS2<br>RASL11A<br>B3GNT5<br>PLOD3<br>RGMB<br>GCA<br>CHRNA10<br>LGALS3BP<br>GPR75<br>DHRS12	ATP6V0A1<br>ZDHHC14<br>C2CD2<br>HSD17B6<br>CD300C<br>**MEGF8**<br>LYZ<br>GRASP<br>MAPK15<br>BTK<br>PLOD3<br>**NTSR1**<br>LAPTM4B<br>TIAM1<br>PGLYRP2<br>CHRNA10<br>**CCR8**<br>KCNH3<br>AIF1<br>LGALS3BP<br>PRRG4<br>**SV2A**<br>FAR2<br>LTK<br>RALGPS2<br>SVIL<br>**CALCRL**<br>PCYOX1L<br>TSSK4<br>MSRB3<br>CYB561D1<br>SLC24A4<br>**EPHB3**<br>IL10<br>TNFSF13B<br>MGLL<br>WDR44<br>LAT2<br>**GPM6B**<br>HSPA13<br>**SEMA6B**<br>**CNTNAP1**<br>IL17C<br>**CTLA4**<br>PPL<br>HOOK1<br>DDN<br>PHLPP1<br>PGAP3<br>JUP

								CD300C<br>HOMER1<br>HSD17B6<br>SRC<br>FCN1<br>COL9A2<br>LTK<br>ACSM3<br>TNFSF13B<br>SPON1<br>SCRIB<br>IL12A<br>SVIL<br>FXYD2<br>BIK<br>ALOX5<br>TIAM1<br>AIF1<br>**DPP4**<br>CDHR1<br>ENOX2<br>**SLC1A2**<br>ACTN1<br>F5<br>CYB561D1<br>PGAP3<br>IMPACT<br>**PLXNA4**<br>DUSP6<br>HSPA13<br>S100A9<br>CHIC1<br>TMEM170B<br>SARNP<br>SEMA6A<br>KCNQ5<br>LYZ<br>**SV2A**<br>SLC38A7<br>LGMN<br>ATP8B3<br>TBXAS1<br>IFT140<br>CCDC136<br>LYSMD4<br>**TTYH3**<br>SPRN<br>CFP<br>JSRP1<br>**PTPRS**	SPRN<br>FCN1<br>**SLC1A2**<br>RASGRF2<br>FITM2<br>TMEM170B<br>S100A9<br>**TTYH3**<br>PRSS22<br>CFP<br>SLC38A7<br>HMOX1

								PPL	
