# Supplementary material for: Transcriptional analysis of peripheral memory T cells reveals Parkinson’s disease-specific gene signatures
Source: NPJ Parkinsons Dis. 2022 Mar 21;8:30. doi: 10.1038/s41531-022-00282-2 (PMC8938520; doi:10.1038/s41531-022-00282-2)
Supplement: Supplementary file 2 — Reporting Summary Checklist [file 41531_2022_282_MOESM2_ESM.pdf]

nature portfolio

Corresponding author(s): Cecilia S. Lindestam Arlehamn
Alessandro Sette

Last updated by author(s): Dec 2, 2021

Reporting Summary

Nature Portfolio wishes to improve the reproducibility of the work that we publish. This form provides structure for consistency and transparency in reporting. For further information on Nature Portfolio policies, see our Editorial Policies and the Editorial Policy Checklist.

Statistics

For all statistical analyses, confirm that the following items are present in the figure legend, table legend, main text, or Methods section.

n/a	Confirmed	
☐	☒	The exact sample size (n) for each experimental group/condition, given as a discrete number and unit of measurement
☐	☒	A statement on whether measurements were taken from distinct samples or whether the same sample was measured repeatedly
☐	☒	The statistical test(s) used AND whether they are one- or two-sided<br>*Only common tests should be described solely by name; describe more complex techniques in the Methods section.*
☐	☒	A description of all covariates tested
☐	☒	A description of any assumptions or corrections, such as tests of normality and adjustment for multiple comparisons
☐	☒	A full description of the statistical parameters including central tendency (e.g. means) or other basic estimates (e.g. regression coefficient) AND variation (e.g. standard deviation) or associated estimates of uncertainty (e.g. confidence intervals)
☐	☒	For null hypothesis testing, the test statistic (e.g. F, t, r) with confidence intervals, effect sizes, degrees of freedom and P value noted<br>*Give P values as exact values whenever suitable.*
☒	☐	For Bayesian analysis, information on the choice of priors and Markov chain Monte Carlo settings
☒	☐	For hierarchical and complex designs, identification of the appropriate level for tests and full reporting of outcomes
☒	☐	Estimates of effect sizes (e.g. Cohen's d, Pearson's r), indicating how they were calculated

Our web collection on statistics for biologists contains articles on many of the points above.

Software and code

Policy information about availability of computer code

Data collection	No software was used.
Data analysis	A more detailed description of the softwares used can be found in the various methods sections. In brief, for RNA-seq analyis the softwares Gencode (v27), STAR (v2.6.1), PRINSEQ Lite (v.0.20.3), SAMtools, featureCounts(v1.6.5), and R/Bioconductor package DESeq2. For other statistical analyses, GraphPadSoftware was used.

For manuscripts utilizing custom algorithms or software that are central to the research but not yet described in published literature, software must be made available to editors and reviewers. We strongly encourage code deposition in a community repository (e.g. GitHub). See the Nature Portfolio guidelines for submitting code & software for further information.

Data

Policy information about availability of data

All manuscripts must include a data availability statement. This statement should provide the following information, where applicable:

- Accession codes, unique identifiers, or web links for publicly available datasets
- A description of any restrictions on data availability
- For clinical datasets or third party data, please ensure that the statement adheres to our policy

Data generated or analyzed during this study are included in this article and its Supplementary Information. The datasets generated during and/or analyzed during the current study are available in the Gene Expression Omnibus under accession number GSE174473 (http://www.ncbi.nlm.nih.gov/geo/). Other datasets are available through the Zenodo data depository FCS: 10.5281/zenodo.5523274 and 10.5281/zenodo.5248631. Fluorospot: 10.5281/zenodo.5703708. Experimental protocols are available on protocols.io.

Field-specific reporting

Please select the one below that is the best fit for your research. If you are not sure, read the appropriate sections before making your selection.

☒ Life sciences ☐ Behavioural & social sciences ☐ Ecological, evolutionary & environmental sciences

For a reference copy of the document with all sections, see nature.com/documents/nr-reporting-summary-flat.pdf

Life sciences study design

All studies must disclose on these points even when the disclosure is negative.

Sample size	No sample size calculation was performed since we did not have an expected frequency of response. The final sample size used was based on the samples available from the recruitment sites.
Data exclusions	Data was excluded from Fluorospot analysis according to predefined criteria of either cell death in culture preventing further experiments, or a failed PHA response in the assay (<100 SFC per million cells).<br>Quality control measures were determined at the start of the study to apply to the data generated. For RNA-seq it included removing absent features as well as setting a cutoff for our results (>1.5 differential expression, padj<0.05)
Replication	Our study included a validation cohort to confirm some of the results that we found in our first cohort. Each individual sample was tested in one experiment.
Randomization	Participants were allocated into experimental groups based on PD diagnosis or HC. They were further divided based on having immune reactivity against alpha-synuclein, as measured by Fluorospot.
Blinding	Individuals performing the experiments and collecting the raw data were blinded to cohort assignment PD vs HC. Individuals analyzing the data were not blinded, and performed the subdivision into PD-R, PD-NR and HC-NR, since the comparisons were dependent on knowing the respective cohort.

Reporting for specific materials, systems and methods

We require information from authors about some types of materials, experimental systems and methods used in many studies. Here, indicate whether each material, system or method listed is relevant to your study. If you are not sure if a list item applies to your research, read the appropriate section before selecting a response.

Materials & experimental systems

n/a	Involved in the study	
☐	☒	Antibodies
☒	☐	Eukaryotic cell lines
☒	☐	Palaeontology and archaeology
☒	☐	Animals and other organisms
☐	☒	Human research participants
☒	☐	Clinical data
☒	☐	Dual use research of concern

Methods

n/a	Involved in the study	
☒	☐	ChIP-seq
☐	☒	Flow cytometry
☒	☐	MRI-based neuroimaging

Antibodies

Antibodies used	APCef780 conjugated anti-CD4 (clone RPA-T4, eBiosciences, RRID:AB_1272044), AF700 conjugated anti-CD3 (clone UCHT1, BD Pharmigen, RRID:AB_10597906), BV650 conjugated anti-CD8a (clone RPA-T8, Biolegend, RRID:AB_11125174), PECy7 conjugated anti-CD19 (clone HIB19, TONBO, RRID:AB_2621841), APC conjugated anti-CD14 (clone 61D3, TONBO, RRID:AB_2621560), PerCPCy5.5 conjugated anti-CCR7 (clone G043H7, Biolegend, RRID:AB_10916121), PE conjugated anti-CD56 (eBiosciences, RRID:AB_10598200), FITC conjugated anti-CD25 (clone M-A251, BD Pharmigen, RRID:AB_395825), eF450 conjugated anti-CD45RA (clone HI100, eBiosciences, RRID:AB_1272059) and eF506 live dead aqua dye (eBiosciences, 65-0866-1). AF700 conjugated anti-CD3 (clone UCHT1, BD Pharmigen, RRID:AB_10597906), BV650 conjugated anti-CD8a (clone RPA-T8, Biolegend, RRID:AB_11125174), eF450 conjugated anti-CD45RA (clone HI100, eBiosciences, RRID:AB_1272059), PerCPCy5.5 conjugated anti-CCR7 (clone G043H7, Biolegend, RRID:AB_10916121), BV786 conjugated anti-CD4 (clone SK3, BD Biosciences, RRID:AB_2738462), FITC conjugated anti-CD26 (clone BA5b, BioLegend, RRID:AB_314288), PECy7 conjugated anti-CCR1 (clone 5F10B29, BioLegend, RRID:AB_2734400), PE conjugated anti-CX3CR1 (clone 2A9-1, BioLegend, RRID:AB_1595456), BV605 conjugated anti-CCR5 (clone 2D7/CCR5, BD Biosciences, RRID:AB_2738167), PE-CF594 conjugated anti-CTLA-4 (clone BNI3, BD Biosciences, RRID:AB_2737761), APC-Cy7 conjugated anti-CD36 (clone 5-271, BioLegend, RRID:AB_2072512), and eF506 live dead aqua dye (eBiosciences, 65-0866-1). mouse anti-human IFN (clone 1-D1K, RRID:AB_907283), mouse anti-human IL-5 (clone TRFK5, RRID:AB_907349), and mouse anti-human IL-10 (clone 9D7, RRID:AB_907307). IFN (7-B6-1-FS-BAM), IL-5 (5A10-WASP), and IL-10 (12G8-biotin). anti-BAM-490, anti-WASP-640, and SA-550—RRID:AB_907273, RRID:AB_907353, RRID:AB_907309.

Validation | All antibodies used were tested and optimized in preliminary studies in the same panel they were used in for the eventual publication data experiments.

Human research participants

Policy information about studies involving human research participants

Population characteristics	PD patients were enrolled on the basis of the following criteria: moderate to advanced PD; 2 of: rest tremor, rigidity, and/or bradykinesia, PD diagnosis at age 45-80, dopaminergic medication benefit, and ability to provide informed consent. The exclusion criteria were atypical parkinsonism or other neurological disorders, history of cancer within past 3 years, autoimmune disease, and chronic immune modulatory therapy. Age matched HC were selected on the basis of age 45-85 and ability to provide written consent. Exclusion criteria were the same as for PD donors and in addition, we excluded self-reported genetic factors. The HC were not screened for prodromal symptoms. The PD patients recruited at RUMC, UAB, CUMC, and UCSD (i.e. not at LJI) all fulfilled the UK Parkinson's Disease Society Brain Bank criteria for PD.
Recruitment	The cohorts were recruited by the clinical core at LJI, by the Parkinson and Other Movement Disorder Center at UCSD, the clinical practice of the UAB Movement Disorders Clinic, and the Movement Disorders Clinic at the department of Neurology at CUMC. Potential bias in recruitment is the partner's of individuals with PD being over-represented--contributing to a gender gap between the clinical cohorts.
Ethics oversight	All participants provided written informed consent for participation in the study. Ethical approval was obtained from the Institutional review boards at La Jolla Institute for Immunology (LJI; Protocol Nos: VD-124 and VD-118), Columbia University Medical Center (CUMC; protocol number IRB-AAAQ9714 and AAAS1669), University of California San Diego (UCSD; protocol number 161224), Rush University Medical Center (RUMC; Office of Research Affairs No.16042107-IRB01) and the University of Alabama at Birmingham (UAB; protocol number IRB-300001297).

Note that full information on the approval of the study protocol must also be provided in the manuscript.

Flow Cytometry

Plots

Confirm that:

☒ The axis labels state the marker and fluorochrome used (e.g. CD4-FITC).

☒ The axis scales are clearly visible. Include numbers along axes only for bottom left plot of group (a 'group' is an analysis of identical markers).

☒ All plots are contour plots with outliers or pseudocolor plots.

☒ A numerical value for number of cells or percentage (with statistics) is provided.

Methodology

Sample preparation	The cryopreserved PBMC were thawed and revived in prewarmed RPMI media supplemented with 5% human serum (Gemini Bio-Products, West Sacramento, CA), 1 % Glutamax (Gibco, Waltham, MA), 1% penicillin/streptomycin (Omega Scientific, Tarzana, CA), and 50 U/ml Benzonase (Millipore Sigma, Burlington, MA). The cells were then counted using a hemocytometer, washed with PBS and prepared for staining. The cells at a density of 1 million were first incubated at 4[illegible]C with 10% FBS for 10 mins for blocking and then stained with a mixture of antibodies.
Instrument	BD FACSAria and BD LSRFortessa
Software	FlowJo version 10
Cell population abundance	100,000 cells of single, live, CD4, CD8, and PBMCs were counted for RNA analysis. 1million live PBMCs were used to stain for validation flow cytometry experiments.
Gating strategy	Cells were gated on appropriate PBMC FSC-SSC and then subsequently gated to exclude singlets (FSC-H/FSC-A and SSC-H/SSC-A). Cells that excluded our viability dye were then gated on appropriate T cell populations (positivity determined by the prescence of discrete low and high populations).

☒ Tick this box to confirm that a figure exemplifying the gating strategy is provided in the Supplementary Information.
